# False Positive Findings of [^18^F]PSMA-1007 PET/CT in Patients After Radical Prostatectomy with Undetectable Serum PSA Levels

**DOI:** 10.3389/fsurg.2022.943760

**Published:** 2022-06-24

**Authors:** Marina Orevi, Simona Ben-Haim, Galith Abourbeh, Alexandre Chicheportiche, Eyal Mishani, Vladimir Yutkin, Ofer N. Gofrit

**Affiliations:** ^1^ Department of Nuclear Medicine and Medical Biophysics, Faculty of Medicine, Hebrew University of Jerusalem, Hadassah Medical Center, Jerusalem, Israel; ^2^ University College London and UCL Hospitals, NHS Trust, London, United Kingdom; ^3^ Cyclotron/Radiochemistry Unit, Hadassah Medical Center, Jerusalem, Israel; ^4^ Department of Urology, Faculty of Medicine, Hebrew University of Jerusalem, Hadassah Medical Center, Jerusalem, Israel

**Keywords:** prostate cancer, [^18^F]PSMA-1007 PET/CT, undetectable serum PSA levels, false positive, cancer

## Abstract

**Background:**

PET-CT using prostate-specific membrane antigen (PSMA)-targeting radiopharmaceuticals labeled with ^68^Ga or ^18^F has emerged as the most sensitive staging tool in prostate cancer (PC). Nonetheless, the occurrence of false positive (FP) findings presents a major concern of this approach. In this prospective study, we investigated the frequency and pattern of false-positive findings of [^18^F]PSMA-1007 PET/CT in patients after radical prostatectomy with undetectable serum PSA levels. Any discrete non-physiological accumulation of [^18^F]PSMA-1007 in this population is by definition FP.

**Methods:**

Seventeen men after radical prostatectomy, whose serum PSA levels were <0.05 ng/mL at 2–24 months after surgery were prospectively recruited. PET/CT was acquired at both 1 and 2 h after injection of [^18^F]PSMA-1007.

**Findings:**

Three studies (18%) were interpreted as completely normal. Thirty-five foci of “non-physiological” uptake were observed in the remaining 14 (82%) patients, including a single skeletal focus in four patients, multiple skeletal foci in five patients and soft tissue uptake in eight, including in a desmoid tumor and in pelvic lymphocele. The SUV_max_ of all lesions was in the range of 1–7, except for the desmoid tumor which measured 12.7. All foci were visible in both the 1- and the 2 h studies, presenting a minor (<10%), statistically insignificant increase of SUV_max_ during this time-interval.

**Interpretation:**

FP [^18^F]PSMA-1007-avid foci are found in about 80% of patients with undetectable serum PSA levels. Thus, focal uptake of [^18^F]PSMA-1007 outside its physiological distribution is not a categorical sign of metastasis and can arise from non-specific uptake of the ligand. The interpretation of [^18^F]PSMA-1007 PET/CT studies should always consider the clinical context, and lesions with SUV_max_ < 7 are suspicious for FP.

## Introduction

PET/CT with PSMA-targeted radiopharmaceuticals has evolved into a leading imaging modality in the staging and restaging of prostate cancer (PC). Recent studies have shown that it is a sensitive diagnostic tool for both initial detection and staging of PC and for locating recurrence, changing the management of approximately half of the patients (1, 2). Compared to other radiopharmaceuticals, such as radiolabeled choline or [^18^F]fluciclovine, PSMA-targeted ligands present higher target to background ratios, higher sensitivity (0.65–0.92), specificity (0.84–0.97) and inter-reader agreement (3).

Several PSMA-targeted PET pharmaceuticals are clinically employed, including [^18^F]DCFPyL, [^68^Ga]PSMA-11 and [^18^F]PSMA-1007. The latter offers several advantages, such as the cyclotron-production of fluorine-18, a convenient physical half-life (∼110 min) allowing central distribution, and a relatively low positron energy compared to ^68^Ga, which contributes to an improved spatial resolution. Notably, [^18^F]PSMA-1007 is not excreted in the urine, thereby facilitating the detection of local recurrence.

In a prospective head-to-head comparison of [^68^Ga]PSMA-11 and [^18^F]PSMA-1007 in patients with primary disease, the two pharmaceuticals showed an almost perfect concordance (Cohen k-coefficient range 0.871-1) in detection of the dominant prostatic lesion with significantly higher SUV_max_ (*p* = 0.002) and superiority of [^18^F]PSMA-1007 in detecting additional low-grade lesions (4). [^18^F]PSMA-1007 has also demonstrated a remarkable sensitivity for detecting lymph node metastases, identifying 18/19 involved lymph nodes, some of them as small as 1 mm in diameter (5). In the recently published large prospective randomized proPSMA trial, the accuracy of [^68^Ga]PSMA-11 PET was compared to that of conventional imaging with CT and bone scan in the pretreatment setting. PET-PSMA had a 37% greater accuracy in detecting metastases and changed the management of 23% of the patients compared to 5% with conventional imaging. The authors concluded that PET-PSMA is a suitable replacement for conventional imaging (6). Having said that, verification using hard criteria was employed in only 23% of patients with PSMA-positive lesions; raising the concern that some lesions might have been false-positively interpreted (7). Reports on [^18^F]PSMA-1007 in the post-treatment setting depict an even more complex situation, wherein tumor recurrence was visualized in 81% of the patients with rising PSA. Of interest, in almost 53% of patients with serum PSA levels as low as 1–2 ng/mL there were “findings indicating of bone metastases” (8). In the clinical setting, bone metastases are usually accompanied by much higher levels of PSA, with a mean serum PSA of 147 and 162 ng/mL in two recent reports (9, 10). Moreover, in a matched-pair comparison of [^68^Ga]PSMA-11 and [^18^F]PSMA-1007 in PC patients with biochemical recurrence after radical prostatectomy (RP), Rauscher and colleagues reported that [^18^F]PSMA-1007 had detected roughly 5-times more lesions attributed to benign origin than [^68^Ga]PSMA-11 (11). Thus, the issue of FP findings using [^18^F]PSMA-1007 merits further investigation (12).

Additionally, there are controversies concerning the optimal timing for [^18^F]PSMA-1007 PET acquisitions. Published literature recommends performing acquisition two hours after injection, however, due to practical reasons, many institutions scan after one hour (13, 14).

The aim of the present study was to evaluate the prevalence of false positive [^18^F]PSMA-1007 avid foci in a group of patients after RP, whose serum PSA levels were <0.05 ng/mL. In this unique population, any “non-physiological” uptake of the radiopharmaceutical could be considered FP by definition. Scanning was performed twice, one and two hours after [^18^F]PSMA-1007 injection.

## Materials and Methods

### Study Design and Patient Population

Patients with intermediate-unfavorable or high-risk PC, defined as either International Society of Urological Pathology (ISUP) ≥3 on the final pathology or preoperative PSA level ≥20 ng/mL who were ≥2 months after RP with post-operative PSA levels <0.05 ng/mL were prospectively recruited. All patients have signed an informed consent and had PSA levels <0.05 ng/mL confirmed 3 months after the study. The study was approved by the local IRB committee (#HMC-19-0722).

### Radiosynthesis

The automated, one-step synthesis of [^18^F]PSMA-1007 was carried out as previously reported (15). Mean radiochemical purity at the end of synthesis was 96.2 ± 1.6% (range 94.1%–99.4%), with no detectable ^18^F-fluoride in 13/14 syntheses and 1.1% ^18^F^−^ in one synthesis.

### Imaging Protocol

The studies were performed on either a Discovery MI digital PET/CT (*n* = 13) or on a Discovery MI-DR PET/CT (*n* = 4) scanner (GE Healthcare, Milwaukee WI, USA). Low-dose CT was acquired before each PET study for attenuation correction (Smart current: 15–20 mA, noise index: 14.52), followed by a second diagnostic CT acquisition with or without intravenous contrast (Smart current: 100–500 mA, noise index: 15.32).

Two PET studies were performed: the first was acquired at 67 ± 8 min (*n* = 17) and the second at 126 ± 5 min (*n* = 15) after injection of [^18^F]PSMA-1007 (3.4 ± 0.3 MBq/kg). All patients received an iodine-based oral contrast during the uptake period and, when possible, an intravenous contrast (Omnipaque™ 350, GE Healthcare, 1 mL/kg) prior to the diagnostic CT. The second study acquisition was performed with very low dose CT.

### Visual Interpretation and Semi-Quantitative Analysis

All studies were reviewed independently by two experienced nuclear medicine physicians, with over 10 years of experience each, using the Siemens Syngo®.via workstation. The readers were aware of the clinical background. Disagreements were discussed, and a consensus was reached. After visual interpretation, areas of increased accumulation were evaluated semi-quantitatively by measuring the maximal standardized uptake values (SUV_max_ normalized to body weight). Increased uptake was defined as any accumulation above the adjacent background, not compatible with the conventional anatomical or physiological accumulation of the radiopharmaceutical. One hour and two-hour SUV_max_ were compared using Student's *t*-test for paired samples.

## Results

Seventeen patients were recruited. Dual-time PET/CT scans were performed in 15 patients (2 patients refused the second acquisition). Clinical characteristics of the patients are presented in Supplementary Table S1. Mean age was 66.3 years (±6.0, range: 53–74), mean preoperative PSA was 11.9 ng/mL (±10.9 range: 3.4–41). In 8 patients, final ISUP was 3 and in 9 patients it was 4–5. All patients had PSA levels <0.05 ng/mL 3 months after the study confirming no evidence of disease, and obviating the need to biopsy the lesions seen on PET/CT. One patient (#3) had a PSA rise to 0.1 ng/mL, 23 months after the scan. He is currently under surveillance without further treatment. Another patient (#8) had PSA levels of 0.3 ng/mL, 5 months after the study, and was referred to salvage radiotherapy, which he thus far denied. All other patients maintained undetectable PSA levels after the PET/CT. Average follow-up after the study was 10.1 months (S.D. 4.5 months, median 10 months, range: 6–23).

**Figure 1:**
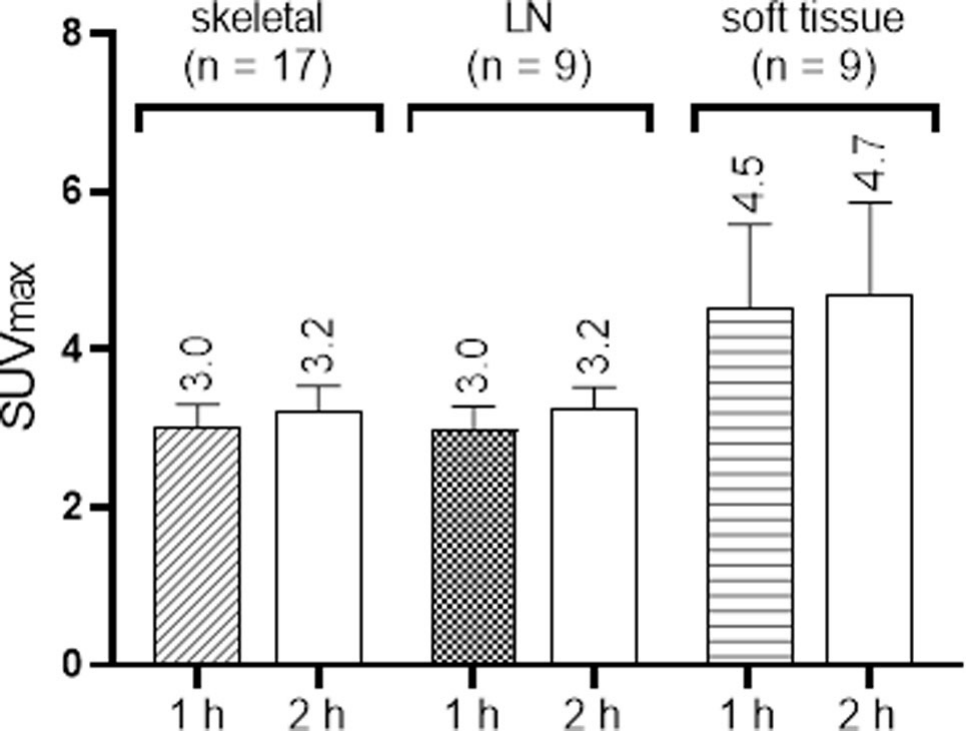
Boxplots of [^18^F]PSMA-1007 uptake (SUV_max_) in different sites 1 and 2 h after injection. Results are presented as mean + SEM.

**Figure 2:**
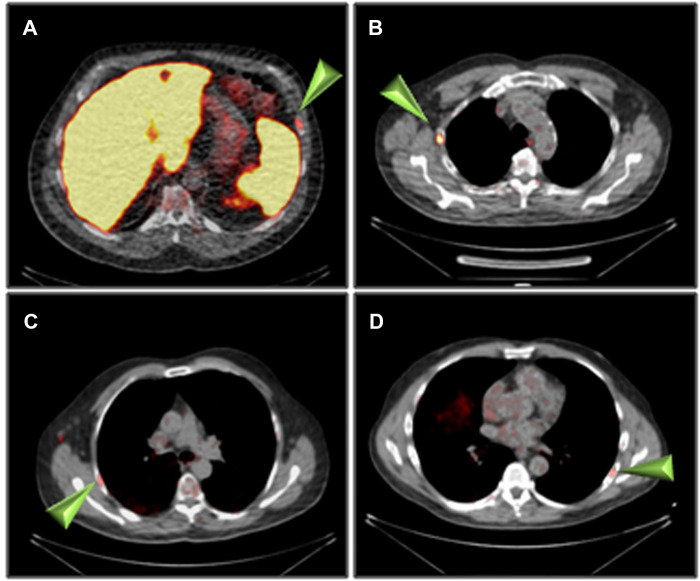
Rib uptake of [^18^F]PSMA-1007 (arrowheads) 2 h after injection. (**A**) patient #4, (**B**) patient #6, (**C**) patient #8, (**D**) patient #11.

**Figure 3:**
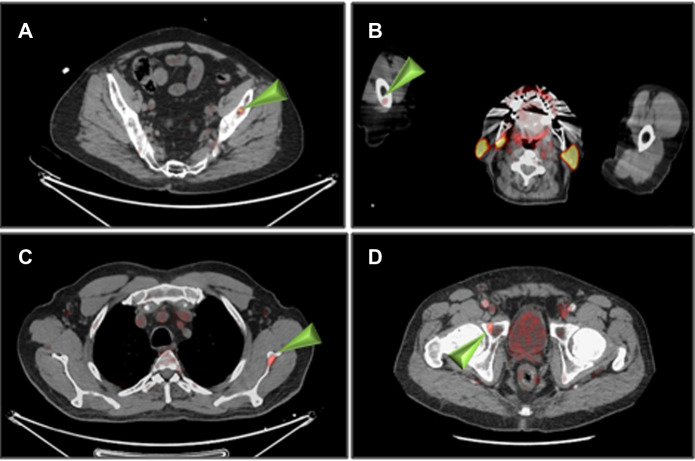
Non-rib skeletal uptake of [^18^F]PSMA-1007 (arrowheads) 1 h after injection. (**A**) patient #1 (left iliac), (**B**) patient #1 (right humerus), (**C**) patient #3 (left scapula), (**D**) patient #10 (right pubic bone).

**Figure 4:**
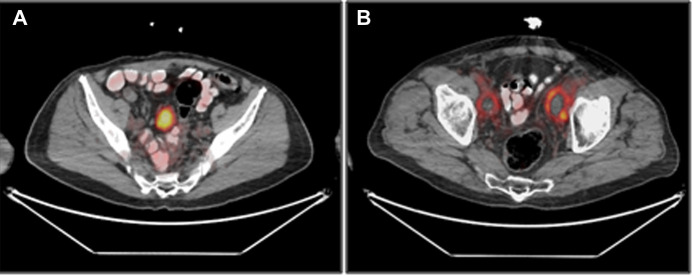
Soft tissue uptake of [^18^F]PSMA-1007, 1 h after injection. (**A**) uptake in a pelvic mass, subsequently identified as a desmoid tumor (patient #9). (**B**) Uptake in bilateral pelvic lymphoceles (patient #13).

The summary of PET/CT findings is presented in Supplementary Table S2 and in Figure. 1. The studies of three patients were normal, whereas in 14 patients, focal uptake was visualized. All foci were visualized in both the 1 h and the 2 h studies with only minor, non-statistically significant differences in the mean SUV_max_. Specifically, the mean ± SD SUV_max_ at 1- and 2 h for skeletal lesions were: 3.0 ± 1.1 and 3.2 ± 1.3, for lymph nodes: 3.0 ± 0.9 and 3.2 ± 0.8 and for soft tissue lesions: 4.5 ± 3.1 and 4.7 ± 3.5, respectively (Supplementary Table S2). The SUV_max_ of all focal non-physiological lesions were in the range of 1.3–11.4 at 1 h after injection (average 3.4, S.D. 1.8, median 2.9) and 1.0–12.7 at 2 h (average 3.6, S.D. 2.1, median 3.1). Patterns of uptake are depicted in Figures 1–4. Four patients had a single skeletal focus of increased [^18^F]PSMA-1007 uptake. These solitary foci included ribs in 2 patients (#4, and #11), the scapula (patient #9) and the pubic bone (patient #15). Multiple skeletal foci were visualized in five patients (#1, #5, #6, #10 and #17), including the ribs, pelvic bones, vertebrae and humerus. Thirteen patients with [^18^F]PSMA-1007-avid skeletal foci had no corresponding abnormality on CT. In two patients, corresponding sclerotic lesions were found in the scapula (#3) and in an osteophyte (#13).

Focal soft tissue uptake was observed in eight patients (#1, #5, #6, #8, #9, #12–14), and included foci in the thyroid, the skin and lymph nodes. One patient (#9) had a 5 cm highly [^18^F]PSMA-1007-avid pelvic mass, (Figure 4A). Laparoscopic resection of the mass revealed a desmoid tumor. Another patient (#13), scanned 4 months after surgery, had bilateral post-surgical pelvic lymphoceles with moderate peripheral [^18^F]PSMA-1007 accumulation (Figure 4B).

## Discussion

The development of urea-based peptidomimetic PSMA radioligands is an important scientific achievement. However, neither is PSMA expression prostate-specific, nor are these ligands, which demonstrate “physiological” uptake in PSMA-negative tissues and organs, such as the liver and the salivary glands (5, 16, 17). In concordance with our findings, uptake of PSMA-ligands has also been reported in a variety of benign conditions associated with osteoblastic activity including osteoarthritis, degenerative changes, fibrous dysplasia, in healing fractures, after radiotherapy and in Paget's disease of bone. Like in the present study, corresponding skeletal CT findings are often absent. Non-PC-specific accumulation of PSMA-radioligands is most likely related to the elevated expression of PSMA in the endothelial cells of the neovasculature, as well as the high permeability of inflammatory cells and macrophage folate receptors (18).

In the clinical setting of PC staging, focal uptake outside the normal distribution of [^18^F]PSMA-1007 is suspicious of metastatic disease, and the validation of such finding is not always done. For example, in the proPSMA trial, only 23% of the focal findings were validated (6). The gold-standard validation is by histology; however, it is often impossible or exceedingly difficult to biopsy a suspected lesion. An indirect validation, by PSA decline following targeted therapy, is also often not addressed (8). Therefore, whether a focal uptake represents a true or false-positive finding remains unanswered in many studies.

In the present study, we investigated this issue from a different angle, by assessing [^18^F]PSMA-1007 uptake in men after RP with undetectable serum PSA levels. To the best of our knowledge, this is the first prospective study using this methodology. Focal uptake in this population would be considered FP by standard definition. Men with high-risk or intermediate-unfavorable risk PC were selected as they can potentially benefit from this baseline evaluation in the event of an eventual PSA rise.

Focal non-physiological uptake was observed in the present study in 82.4% of the patients and was most common in ribs (41.2%) and pelvis (29.4%) (Supplementary Table S2 and Figure. 3). Only 2 patients had corresponding CT findings. Chen et al. have reported that 98.4% of the solitary [^68^Ga]PSMA-11-avid rib foci were benign (19). In a recent retrospective study, the rate of non-specific bone lesions (NSBLs) among PC patients in [^18^F]PSMA-1007 PET/CT was analyzed (20). At least one NSBL was found in 43.9% of patients. Similar to the present study, NSBL were most frequently seen in the ribs (61.3% and 57.5%, respectively), followed by the pelvis (20.1% and 24.8%, respectively) and the spine (11.6%).

A plausible explanation to these skeletal foci, referred to as “non-specific” or “non–PSMA-related”, is lacking. Potential etiologies include all the aforementioned bone-related pathologies, which are associated with osseous remodeling. Grünig et al. observed that NSBLs were more frequent in studies performed with digital PET/CT scanners (70.1%) than with analog ones (40.7%), yet no association was demonstrated with PSA levels, ISUP group, tumor size, age or injected dose (17).

Soft tissue uptake was observed in eight patients (Supplementary Table S2 and Figure. 4A), including lymph nodes (35%) (with non-specific CT appearances) and thyroid (11.7%). One patient had a desmoid tumor, previously reported to accumulate [^68^Ga]PSMA-11 (21). A desmoid tumor should be considered when a discrete soft tissue mass with high uptake of a PSMA radioligand is observed on PET/CT in appropriate locations. In addition, [^18^F]PSMA-1007 accumulation was also documented in the walls of a lymphocele in a study performed 4 months after surgery (Figure. 4A). Skin uptake in multiple locations was noticed in two patients with no specific corresponding abnormality. Uptake of [^18^F]PSMA-1007 was previously reported in several skin conditions including neurofibromatosis, melanoma and angiolipoma, mostly associated with PSMA expression in endothelial capillaries (22–24).

Arnfield et al., suggested that lesions with SUV_max_ < 7.2 are likely benign (20). The current study is in accordance with this observation. The (2 h) SUV_max_ of all lesions was in the range of 1–7, except for the desmoid tumor which measured 12.7. This supports the concept that in the appropriate clinical context lesions with SUV_max_ < 7 are suspicious for being FP.

All focal false-negative lesions were visualized both in the 1- and the 2 h studies with minor changes in SUV_max_. This contrasts with the findings of Rahbar et al. reporting an increase of the median SUV_max_ of [^18^F]PSMA-1007 in PC-lesions by 41.2% between 60 and 120 min (14). We propose a 60-minute uptake time for [^18^F]PSMA-1007, as in most conventional tracers.

The present study findings raise thoughts regarding the soundness of PET-PSMA image interpretations. For example, in a large study by Fendler et al., 653 patients with biochemical failure after RP or radiotherapy were evaluated with [^68^Ga]PSMA-11 PET/CT (25). PET-positive results (determined by a vote of three experts) were found in 75% of the patients. These included prostatic bed, pelvic nodes, and extra-pelvic non-bony and bony findings at different ratios according to the PSA levels. Histologic validation of the findings was available however, in only 87 cases (13.3%) and composite reference standard (i.e., PSA decline after targeted therapy) in 217 cases (33.2%). The current work, as do some of the aforementioned studies (8, 11, 23), suggest that at least some of these foci were not PC metastases and we believe that the information presented in the current study can potentially change the way PSMA-PET is interpreted. Additionally, different populations (patients with rising PSA after treatment in the study by Fendler et al. in contrast to patients with undetectable PSA levels in the current study) and different radiopharmaceuticals ([^68^Ga]PSMA-11 in the study by Fendler et al. study and [^18^F]PSMA-1007 in the current study) may also contribute to the differences.

The limitations of the study are the small sample size and being single centered. However, this is a prospective study in a homogeneous group of patients following RP with no detectable PSA levels and the present study findings support the recently published retrospective data. The theoretical possibility that some of the findings showing focal uptake may represent true positive lesions, i.e. [^18^F]-PSMA-1007 PET/CT is more sensitive than serum PSA for early detection of biochemical failure cannot be completely excluded. It is, however, unlikely since all patients had persistently undetectable levels of PSA for at least 3 months after the study, and only two patients had subsequent minor rises in PSA (after a median follow-up of 10 months).

## Conclusion

False positive [^18^F]PSMA-1007-avid foci were observed in over 80% of men after radical prostatectomy, who had undetectable PSA levels. These foci were mainly seen in the skeleton, most commonly in the ribs and pelvis and almost all of them had SUV_max_ < 7. Awareness to this potential pitfall is of paramount importance during the interpretation of [^18^F]PSMA-1007 PET/CT studies, to avoid misinterpretation and unnecessary diagnostic procedures. The interpretation of [^18^F]PSMA-1007 PET/CT studies should always consider the clinical context, and lesions with SUV_max_ < 7 must be suspicious for FP.

## Data Availability

The original contributions presented in the study are included in the article/Supplementary Material, further inquiries can be directed to the corresponding author/s.
