# Supplementary material for: False Positive Findings of [18F]PSMA-1007 PET/CT in Patients After Radical Prostatectomy with Undetectable Serum PSA Levels
Source: Front Surg. 2022 Jun 24;9:943760. doi: 10.3389/fsurg.2022.943760 (PMC9263625; doi:10.3389/fsurg.2022.943760)
Supplement: Supplementary file 1 [file Table_1_v1.docx]

**Supplementary Material 1. Patients' pre-operative parameters and final pathological findings**

| No | Age | Pre-operative PSA [ng/mL] | Final ISUP | Time between RP and PET (months) |
| --- | --- | --- | --- | --- |
| 1 | 74 | 41 | 5 | 27 |
| 2 | 64 | 7.3 | 4 | 2 |
| 3 | 67 | 5.1 | 4 | 5 |
| 4 | 66 | 4.7 | 5 | 11 |
| 5 | 68 | 7.1 | 4 | 35 |
| 6 | 73 | 36 | 5 | 49 |
| 7 | 60 | 6.7 | 5 | 3 |
| 8* | 70 | 13 | 3 | 7 |
| 9 | 68 | 8.3 | 3 | 14 |
| 10 | 68 | 8.6 | 3 | 6 |
| 11 | 61 | 5.1 | 3 | 7 |
| 12 | 65 | 18 | 3 | 9 |
| 13 | 74 | 5 | 4 | 4 |
| 14 | 53 | 17 | 3 | 2 |
| 15 | 69 | 5.3 | 4 | 10 |
| 16 | 56 | 9.9 | 3 | 22 |
| 17 | 71 | 3.4 | 3 | 4 |

*The serum PSA of this patient rose to 0.2 ng/mL, 5 months after the [^18^F]PSMA-1007 scan.
