# Supplementary material for: False Positive Findings of [18F]PSMA-1007 PET/CT in Patients After Radical Prostatectomy with Undetectable Serum PSA Levels
Source: Front Surg. 2022 Jun 24;9:943760. doi: 10.3389/fsurg.2022.943760 (PMC9263625; doi:10.3389/fsurg.2022.943760)
Supplement: Supplementary file 2 [file Table_2_v1.docx]

**Supplementary Table 2.** [^18^F]PSMA-1007 PET/CT findings and their SUV_max_ at 1 h and 2 h after injection

|  | Location | 1 h SUV_max_ | 2 h SUV_max_ | CT Findings |
| --- | --- | --- | --- | --- |
| 1 | Lt. iliac  Rt. rib  Lt. rib  Rt. humerus  Rt. hilum LN  Subcarinal LN | 3.4  2.3  2.6  1.5  2.6  3.5 | 4.7  2.8  3.1  2.3  2.9  3.3 |  |
| 2 | Negative study |  |  |  |
| 3 | Lt. scapula | 2.9 | 3.6 | sclerotic lesion |
| 4 | Lt. rib | 2.8 | 3.4 |  |
| 5 | Multiple skin lesions  Lt. iliac  L3  L4 | 3.3, 1.5, 2.1, 4.2  2.6  3.5  4.1 | 4.0, 1.0, 2.5, 3.8  2.2  2.9  3.9 |  |
| 6 | Rt. Iliac crest  Rt. rib  Rt. thyroid  Axillary LN | 2.3  6.5  6.8  1.3 | 2.0  7.0  6.0  2.0 |  |
| 7 | negative study |  |  |  |
| 8 | Rt. rib  Rt. ext. iliac LN  Lt. axillary LN  Rt. cervical LN  Lt. thyroid | 2.4  3.6  2.5  4.2  5.5 | 2.2  3.5  2.6  4.4  5.4 |  |
| 9 | Desmoid tumor  Lt. scapula | 11.4  3.2 | 12.7  1.9 |  |
| 10^*^ | Rt. pubis  Bil. ribs | 4.7  2.5 | N.A. |  |
| 11 | Lt. rib | 2.5 | 2.5 |  |
| 12 | Lt. ext. iliac LN | 2.5 | 3.5 |  |
| 13 | Rt. hilum  Bil. lymphocele  L3 vertebra | 3.9  4.5  4.2 | 4.5  5.4  4.8 | osteophyte |
| 14 | Lt. flank subcutis  Lt. cervical LN | 1.6  2.7 | 1.6  2.5 |  |
| 15 | Lt. pubis | 3.5 | N.A. |  |
| 16 | Negative study |  |  |  |
| 17 | Lt. rib  T7 | 2.6  2.1 | 2.6  2.9 |  |

Lt. = left, Rt. = right, Bil. = bilateral, LN=lymph nodes, ext. = external, N.A.: 2 h scan was not performed.
